# MMP7 interacts with ARF in nucleus to potentiate tumor microenvironments for prostate cancer progression *in vivo*

**DOI:** 10.18632/oncotarget.10251

**Published:** 2016-06-23

**Authors:** Yingqiu Xie, Wenfu Lu, Shenji Liu, Qing Yang, J. Shawn Goodwin, Sandeep Anantha Sathyanarayana, Siddharth Pratap, Zhenbang Chen

**Affiliations:** ^1^ Department of Biology, School of Science and Technology, Nazarbayev University, Astana, 010000, Republic of Kazakhstan; ^2^ Department of Biochemistry and Cancer Biology, Meharry Medical College, Nashville, TN, 37208, USA; ^3^ Department of Surgery, Meharry Medical College, Nashville, TN, 38208, USA; ^4^ School of Graduate Studies and Research, Meharry Medical College, Nashville, TN, 37208, USA

**Keywords:** MMP7, ARF, tumor microenvironments, prostate cancer

## Abstract

ARF couples with TP53 in a canonical signaling pathway to activate cellular senescence for tumor suppressive function under oncogenic insults. However, the mechanisms on aberrant elevation of ARF in cancers are still poorly understood. We previously showed that ARF (p14^ARF^ in human and p19^Arf^ in mouse) elevation correlates with PTEN loss and stabilizes SLUG to reduce cell adhesion in prostate cancer (PCa). Here we report that ARF is essential for MMP7 expression, E-Cadherin decrease and the anchorage loss to the extracellular matrix (ECM) in PCa *in vitro* and *in vivo*. We found that Mmp7 is aberrantly elevated in cytosol and nucleus of malignant prostate tumors of *Pten/Trp53* mutant mice. Interestingly, p19^Arf^ deficiency strikingly decreases Mmp7 levels but increases E-Cadherin in *Pten/Trp53/p19^Arf^* mice. ARF knockdown markedly reduces MMP7 in human PCa cells. Conversely, tetracycline-inducible expression of ARF increases MMP7 with a decrease of E-Cadherin in PCa cells. Importantly, MMP7 physically binds ARF to show the co-localization in nucleus. Co-expression of MMP7 and ARF promotes cell migration, and MMP7 knockdown decreases wound healing in PCa cells. Furthermore, MMP7 elevation correlates with ARF expression in advanced human PCa. Our findings reveal for the first time that the crosstalk between ARF and MMP7 in nucleus contributes to ECM network in tumor microenvironments *in vivo*, implicating a novel therapeutic target for advanced PCa treatment.

## INTRODUCTION

Prostate cancer (PCa) is the second leading cause of cancer-related deaths among men in Western countries and the emerging threat to men in Asian countries [1, 2]. Aberrations of multiple oncogenic pathways contribute to the initiation and progression of PCa [3–6]. Mutation and deletion of phosphatase and tensin homolog deleted on chromosome 10 (PTEN) are frequently found in human cancers, including PCa [7, 8]. *Pten/Trp53* mutant mice provide us a unique and powerful tool to elucidate potential oncogenic factors of PTEN network. However, complete pictures on the mechanisms leading to the striking features of malignancy are still poorly understood. ARF (p14^ARF^ in human and p19^Arf^ in mouse) elevation is found in PTEN-deficient human PCa [9] and various cancer cell lines [10–12]. ARF is originally identified as an alternative transcript of *ARF-INK4a* locus on human chromosome 9q21 (chromosome 4 in mouse) [13]. Induction of the canonical ARF pathway halts cancer progression through coupling with TP53 to induce cellular senescence and inhibiting ribosomal RNA transcription and processing, response to DNA damage and autophagy initiation [14, 15]. Nevertheless, ARF elevation is associated with the triggering of oncogenic pathways, which in turn results in additional alterations of molecular cascades for cancer progression.

We previously demonstrated that ARF stabilizes SLUG to promote epithelial-mesenchymal transition (EMT) in PCa through degradation of cell adhesion [16]. The ECM, a key factor in cell adhesion and migration, is mainly degraded by matrix metalloproteinases (MMPs) [17]. MMP7, one of the secreted proteolytic enzymes, is associated with invasion and metastasis of cancers including PCa [18–20]. The mechanisms on oncogenic contributions of MMP7 to PCa progression in PTEN-null context still remain unclear. Given that *Pten/Trp53* mice produce aggressive PCa through senescence evasion and p19^Arf^ elevation, we took advantage of this mouse model and bioinformatics approaches to investigate the non-canonical ARF signaling in cancers. Our results revealed a genetic landscape mediated by p19^Arf^ in prostate tumors *in vivo* and further identified a novel ARF-MMP7 pathway in tumor microenvironments.

## RESULTS

### Mmp7 expression is p19^Arf^-dependent oncogenic signaling in *Pten/Trp53* mouse model

**Figure 1:**
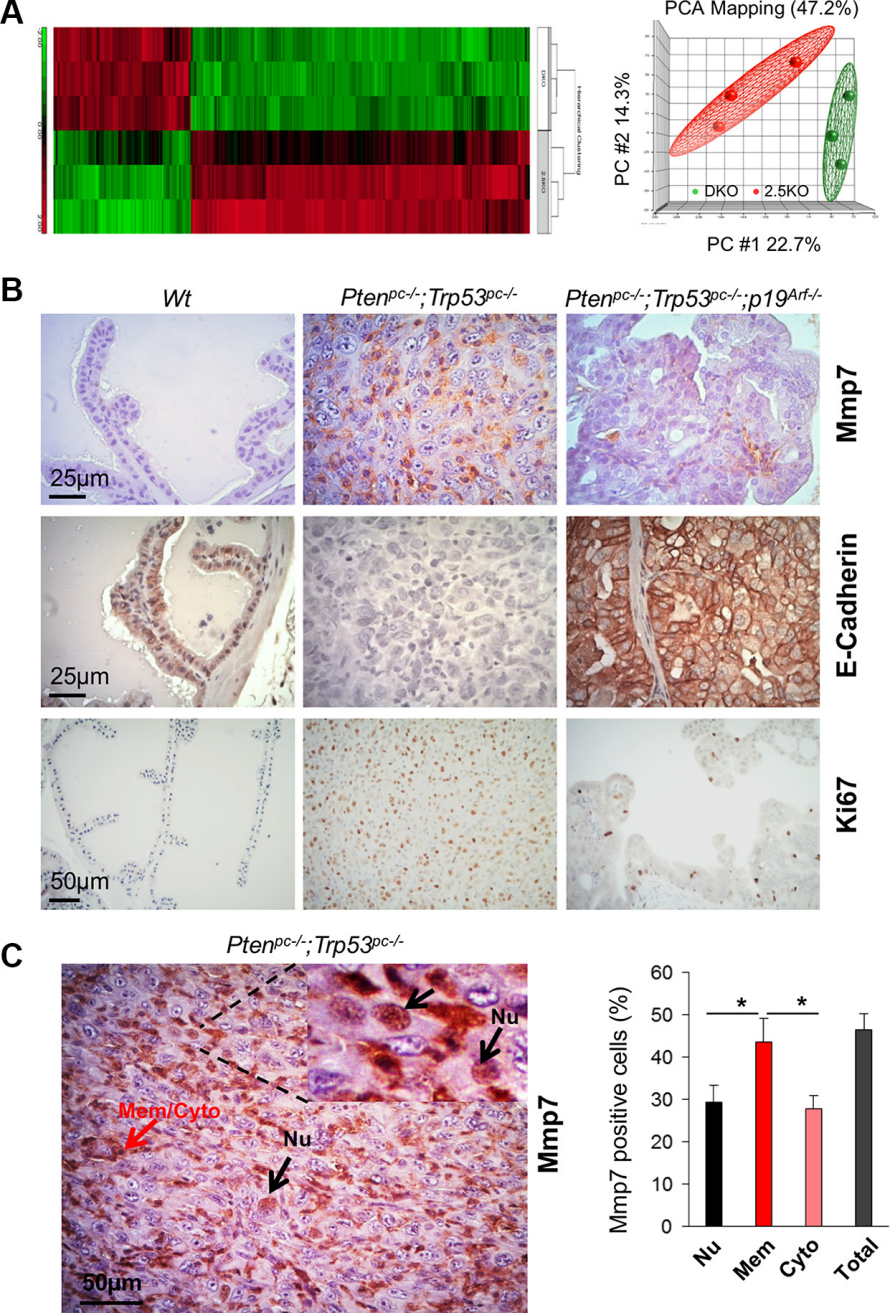
Mmp7 is elevated in prostate cancer of *Pten/Trp53* mice but decreased by loss of p19^Arf^ *in vivo* (**A**) Gene heat map of microarray using RNA extracted from prostate tissues of *Pten*^pc–/−^*;Trp53*^pc–/−^ (DKO), *Pten*^pc–/−^*;Trp53*^pc–/−^*;p19*^Arf+/−^ (2.5KO) mutant mice at 6 months of age. Right panel: data distribution of samples in indicated groups. Please refer Supplementary Tables S1 and S2 for the list of selected genes with fold changes. (**B**) Levels of Mmp7, E-Cadherin and Ki67 in anterior prostates of *Wt, Pten*^pc–/−^*;Trp53*^pc–/−^, and *Pten*^pc–/−^*;Trp53*^pc–/−^*;p19*^Arf–/−^ mutant mice at 6 months of age. (**C**) Mmp7 in nucleus of malignant cells in prostate tumors of *Pten*^pc–/−^*;Trp53*^pc–/−^ mice. Mmp7 expression in nucleus (Nu) (arrows in black color), and Mmp7 at membrane and cytoplasm (Mem/Cyto) (arrows in red color). Right panel: Quantification analysis of cellular localization of Mmp7 expression.

*Pten/Trp53* mutant mice develop aggressive PCa with p19^Arf^ upregulation and loss of epithelial adhesion [16]. In order to gain deep insights into the impact of p19^Arf^ loss on oncogenic pathways in PCa *in vivo*, we applied bioinformatics approaches to investigate the changes of key factors on tumor microenvironments between *Pten/Trp53* and *Pten/Trp53/p19*^Arf^ mutant mice. We performed the transcriptome profiling and histopathology analysis of prostate tumors in *Pten/Trp53* and *Pten/Trp53/p19*^Arf^ mice at 6 months of age (3 mice/group). Our data revealed that p19^Arf^ loss (with one allele deletion of p19^Arf^) resulted in both downregulation and upregulation of genes (Figure 1A), suggesting that p19^Arf^ has biological functions on rRNA formation and protein synthesis, in addition to being a tumor suppressor. p19^Arf^ upregulates significantly a subset of oncogenes such as *Mmp7, Mmp15* and *Muc20,* a MET signaling regulator (Supplementary Tables S1 and S2), at least in the context of *Pten/Trp53* loss. Given that *MMP* genes are primarily involved in ECM and cell adhesion, our results indicate that ARF may activate tumor microenvironments through MMP.

Since Mmp7 was dramatically decreased by p19^Arf^ loss *in vivo*, we decided to investigate the crosstalk between ARF and MMP7 in human PCa cells and to further explore whether ARF increases ECM regulator proteins for PCa progression. Immunohistochemistry (IHC) analysis revealed that levels of Mmp7, Snail and Vimentin were strikingly elevated in *Pten/Trp53* tumors, but were dramatically decreased in *Pten/Trp53/p19*^Arf^ tumors upon p19^Arf^ deficiency (Figure 1B and Supplementary Figure S1). In agreement with the results, p19^Arf^ deficiency increased E-Cadherin and decreased Ki67 in *Pten/Trp53/p19*^Arf^ tumors (Figure 1B). Interestingly, Mmp7 was localized in nucleus of malignant cells in *Pten/Trp53* tumors (Figure 1C), in addition to its localizations at membrane and cytoplasm. Our results support that nuclear MMPs are positively associated with aggressive features of tumors [21], and indicate that a concomitant elevation of MMP7 and ARF in nucleus may be critical for prostate tumorigenesis.

### Nuclear MMP7 is decreased by ARF knockdown in human prostate cancer cells

**Figure 2:**
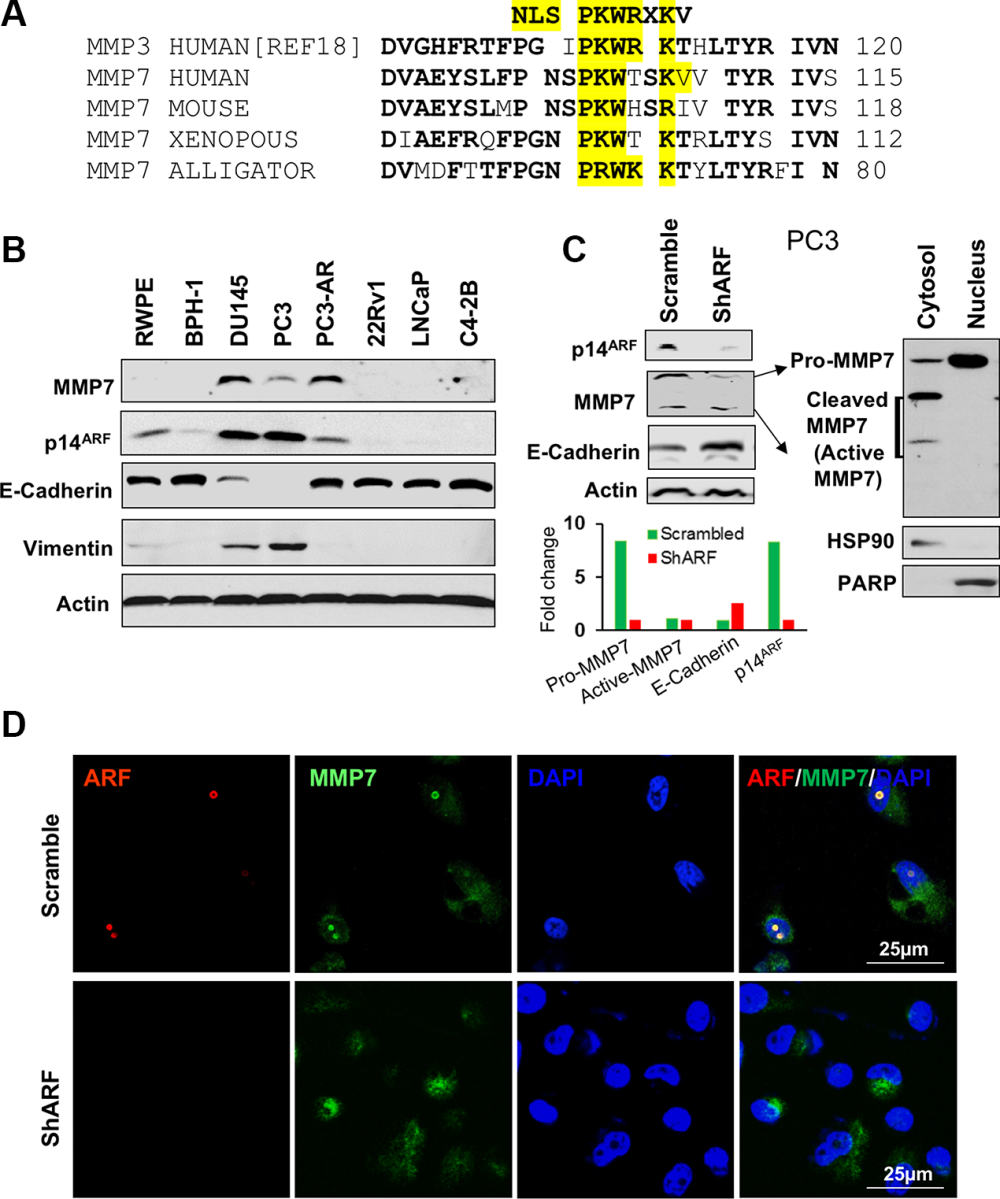
The expression and nuclear localization of MMP7 are decreased by p14^ARF^ knockdown in human prostate cancer cells (**A**) The nucleus localization signal (NLS) is conserved in MMP family among species. (**B**) MMP7 and p14^ARF^ are concomitantly elevated in prostate cancer cells, and MMP7 levels are inversely correlated with E-Cadherin in prostate cancer cells. (**C**) Western blot analysis demonstrates ARF knockdown results in a reduction of MMP7 levels with an increase of E-Cadherin in PC3 cells. MMP7 in nucleus is the pro-form not the active form of MMP7 protein. (**D**) Immunofluorescence (IF) images show the decreased MMP7 in nucleus of PC3 cells upon ARF knockdown.

Our analysis reveals that MMP7 protein contains the PKWXXKV sequence that is partially conserved with the nuclear localization signal (NLS) in MMP3 [18, 22, 23] (Figure 2A), suggesting an alternate mechanism on its shuttling between membrane/cytoplasm to nucleus. Since p19^Arf^ loss decreases Mmp7 in mouse, we reasoned that nuclear MMP7 requires ARF and could be downregulated upon ARF loss in human PCa cells. To test the hypothesis and explore the interactions of MMP7 and ARF, we first examined protein levels of MMP7 and ARF in human PCa and normal cell lines. We found that MMP7 and ARF were highly expressed in PC3 and DU145 PCa cells, while their levels were very low in 22Rv1, LNCaP, C4-2B PCa cells and normal prostate cells. Interestingly, along with the elevation of MMP7 and ARF, levels of Vimentin were increased with a decrease of E-Cadherin in PC3 and DU145 cells (Figure 2B). We chose PC3 cells to knockdown p14^ARF^ by small hairpin RNA interference (shRNA). As confirmed with Western blot, p14^ARF^ knockdown resulted in a striking reduction of MMP7 as compared to the control (Figure 2C). The fractionation analysis revealed that the zymogen pro-form of MMP7 (pro-MMP7), not the active-MMP7, was decreased by p14^ARF^ knockdown. In addition, E-Cadherin was increased upon p14^ARF^ knockdown (Figure 2C), indicating that ARF-MMP7 network determines EMT phenotype in PCa. MMP7 in nucleus consists of the pro-MMP7 only (right panel, Figure 2C), suggesting that pro-MMP7 can shuttle between cytosol and nucleus without cleavage. We then performed immunofluorescence (IF) staining to detect the co-localization of MMP7 and ARF in PC3 and DU145 cells. In agreement with literature, MMP7 protein was localized predominantly at the cytosol and membrane of PC3 cells (Figure 2D). Surprisingly, MMP7 was also aberrantly localized in nucleus and co-localized with ARF in both PC3 and DU145 cells as confirmed with different antibodies (Supplementary Figure S2). p14^ARF^ knockdown abrogated MMP7 in nucleus of PC3 cells at both mRNA and protein levels (Figure 2D, Supplementary Figures S3 and S4). Our results indicate that MMP7 may promote EMT-like signaling through crosstalk with ARF.

### MMP7 is increased with E-Cadherin decrease upon ARF induction in prostate cancer cells

**Figure 3:**
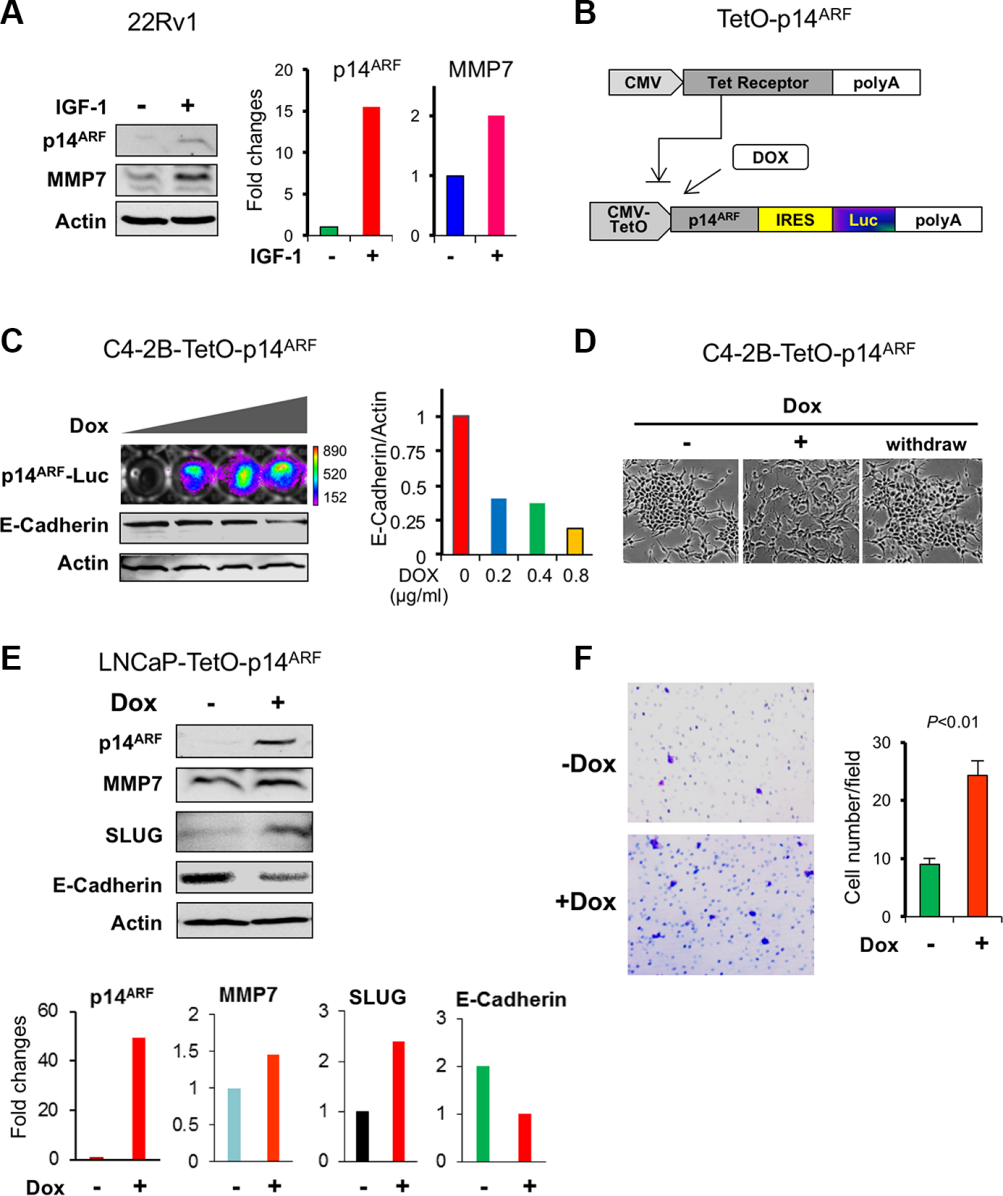
p14^ARF^ overexpression increases MMP7 levels with a decrease of E-Cadherin in human prostate cancer cells (**A**) A concomitant induction of endogenous p14^ARF^ and MMP7 levels by IGF1 (100 μg/ml, 6 hrs) in 22Rv1 PCa cells. (**B**) A schematic diagram of the doxycycline-inducible p14^ARF^ construct. (**C**) p14^ARF^ induction results in a decrease of E-Cadherin in C4-2B cells in a dose-dependent manner. Stable overexpression of p14^ARF^ fused with luciferase reporter gene was induced by doxycycline (Dox) treatment at 2 μg/ml for 2 days. Left panel: Luciferase intensity represented the expression level of p14^ARF^, and E-Cadherin was detected by Western blot. Right panel: quantification of E-Cadherin level. (**D**) The transition of cell morphology in C4-2B cells upon p14^ARF^ induction. (**E**) Changes of MMP7, SLUG, and E-Cadherin in LNCaP cells by p14^ARF^ induction. (**F**) Increased cell migration of LNCaP cells upon p14^ARF^ induction. Migrated cells were counted from three fields and presented as mean values ± s.d. **P < 0.01 indicates the statistical significance by Student's *t*-test (*n* = 3).

We asked ourselves whether ARF upregulation would lead to an increase of MMP7, an opposing effect of p14^ARF^ knockdown on the signaling events in PCa cells. We sought to increase the endogenous levels of ARF in 22Rv1 cells by addition of IGF1. As a result, a concomitant elevation of ARF and MMP7 was indeed observed upon IGF stimulus as compared to the control (Figure 3A). We applied a Tet-On inducible expression system to establish a doxycycline-inducible p14^ARF^ overexpression cell line from C4-2B cells (Figure 3B and 3C). In this system, p14^ARF^ expression driven by a CMV promoter is repressed by Tet receptor but can be activated by addition of doxycycline, as it prevents Tet receptor from binding to the promoter. p14^ARF^ gene is conjugated with luciferase, so p14^ARF^ expression is indicated by luciferase activity in a dose-dependent manner upon addition of doxycycline. Importantly, p14^ARF^ induction resulted in a striking decrease of E-Cadherin and EMT phenotype (Figure 3C). Withdrawal of doxycycline reversed the EMT feature (Figure 3D). Similarly, p14^ARF^ induction increased MMP7 and SLUG with a decrease of E-Cadherin in LNCaP cells (1.5 μg/ml doxycycline, 2 days) (Figure 3E, and panels below for the quantification). Furthermore, p14^ARF^ induction significantly promoted cell migration in LNCaP cells (Figure 3F). Together, our results suggest MMP7 contributes to cell migration and EMT, at least in part through ARF.

### MMP7 binds ARF in nucleus to promote cell migration

**Figure 4:**
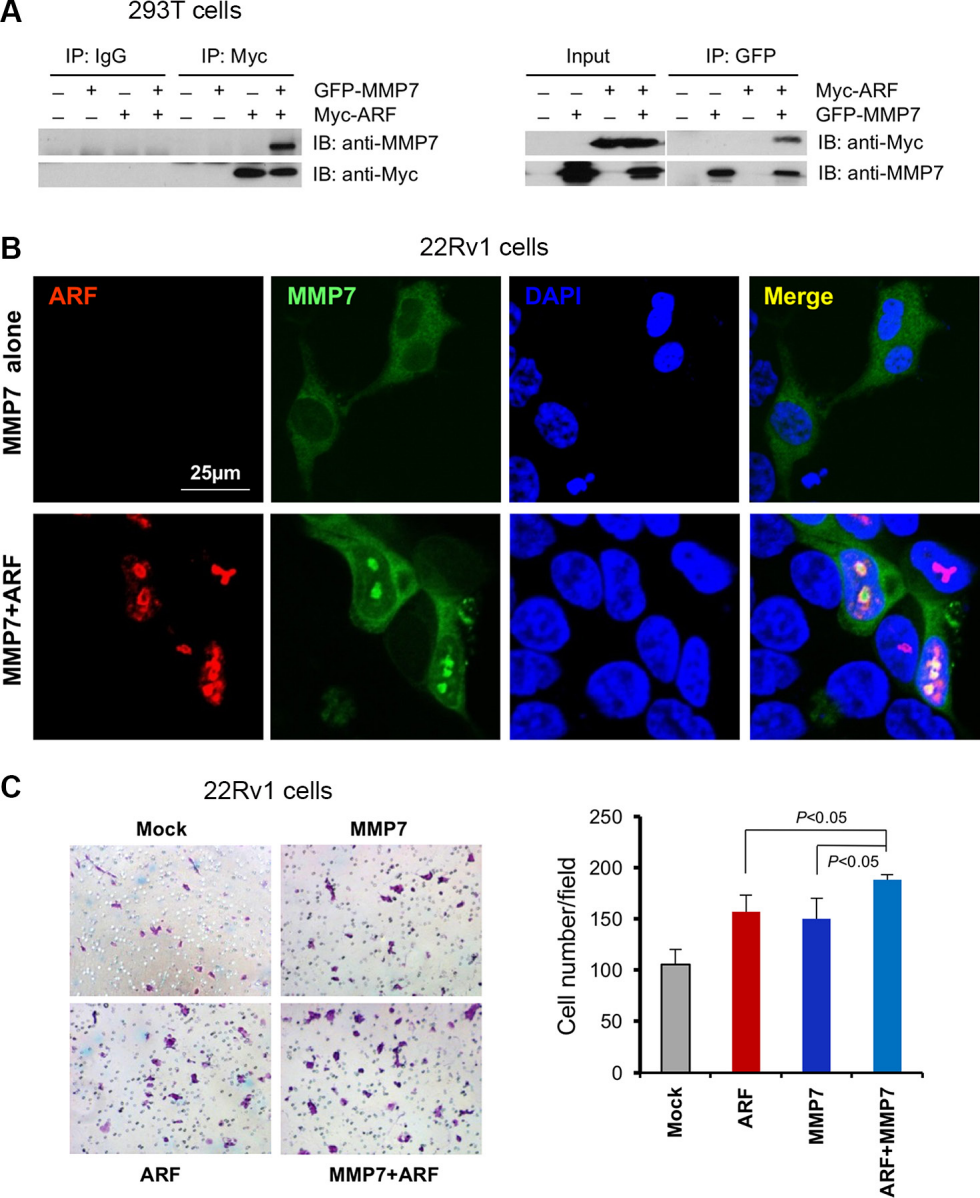
MMP7 interacts with ARF in nucleus to promote cell migration of human prostate cancer cells (**A**) Co-immunoprecipitation of ARF and MMP7 in 293T cells. HEK293T cells were co-transfected with GFP-MMP7 and/or Myc-p14^ARF^ plasmids. Cell lysates were immunoprecipitated with anti-GFP or anti-C-Myc followed with immunoblotting (IB) analysis with indicated antibodies. (**B**) ARF is required for the nuclear localization of MMP7 in 22Rv1 cells. 22Rv1 cells were transfected with GFP-MMP7, Myc-p14^ARF^ or GFP-MMP7 plus Myc-p14^ARF^ plasmids using Lipofectamine 2000 (Invitrogen). Immunofluorescence (IF) images were collected as described previously [16]. (**C**) MMP7 cooperates with ARF to promote cell migration in 22Rv1 cells. 22Rv1 cells transfected with plasmids in (B) were subject to migration analysis. Left panel: the images on migrated cells on the membrane. Right panel: quantification of migrated cells promoted by MMP7 and ARF.

The results above encouraged us to investigate whether MMP7 interacts with ARF to potentiate the oncogenic function for PCa progression. To examine the MMP7-ARF binding, we overexpressed GFP-MMP7 and Myc-p14^ARF^ in HEK293T cells and then performed co-immunoprecipitations (co-IP) with GFP or Myc antibody. Co-IP results demonstrated that MMP7 indeed binds ARF physically, while IgG control samples do not show any binding signals (Figure 4A). As MMP7 is predominantly localized at membrane and cytoplasmic compartments of cells such as Golgi [24], the aberrant interactions of MMP7 and ARF in nucleus propelled us to understand the promotion on PCa progression. We overexpressed MMP7 and p14^ARF^ in 22Rv1 PCa cells that have the undetectable levels of MMP7 and ARF. As predicted, MMP7 protein was indeed co-localized with p14^ARF^ in the nucleus of 22Rv1 cells, while MMP7 alone was exclusively limited to cytosol and membrane (Figure 4B). These data further supported that p14^ARF^ is required for nuclear MMP7, in agreement with the results from p14^ARF^ knockdown (Figure 2C and 2D). We investigated the synergistic effects of MMP7 and ARF overexpression on cell migration. As shown, the combined overexpression of MMP7 and p14^ARF^ significantly increased cell migration of 22Rv1 cells as compared to the control (Figure 4C). Moreover, MMP7 knockdown decreased the wound healing of PC3 cells (Supplementary Figure S5). As ARF is a marker of nucleolus [25, 26], these lines of evidence implicate that MMP7 binding to ARF in nucleolus may contribute to rRNA biogenesis and protein synthesis in PCa cells. Our results indicate that ARF potentiates the oncogenic functions of MMP7 in nucleus for PCa progression.

### MMP7 correlates with ARF expression levels in human prostate cancer specimens

**Figure 5:**
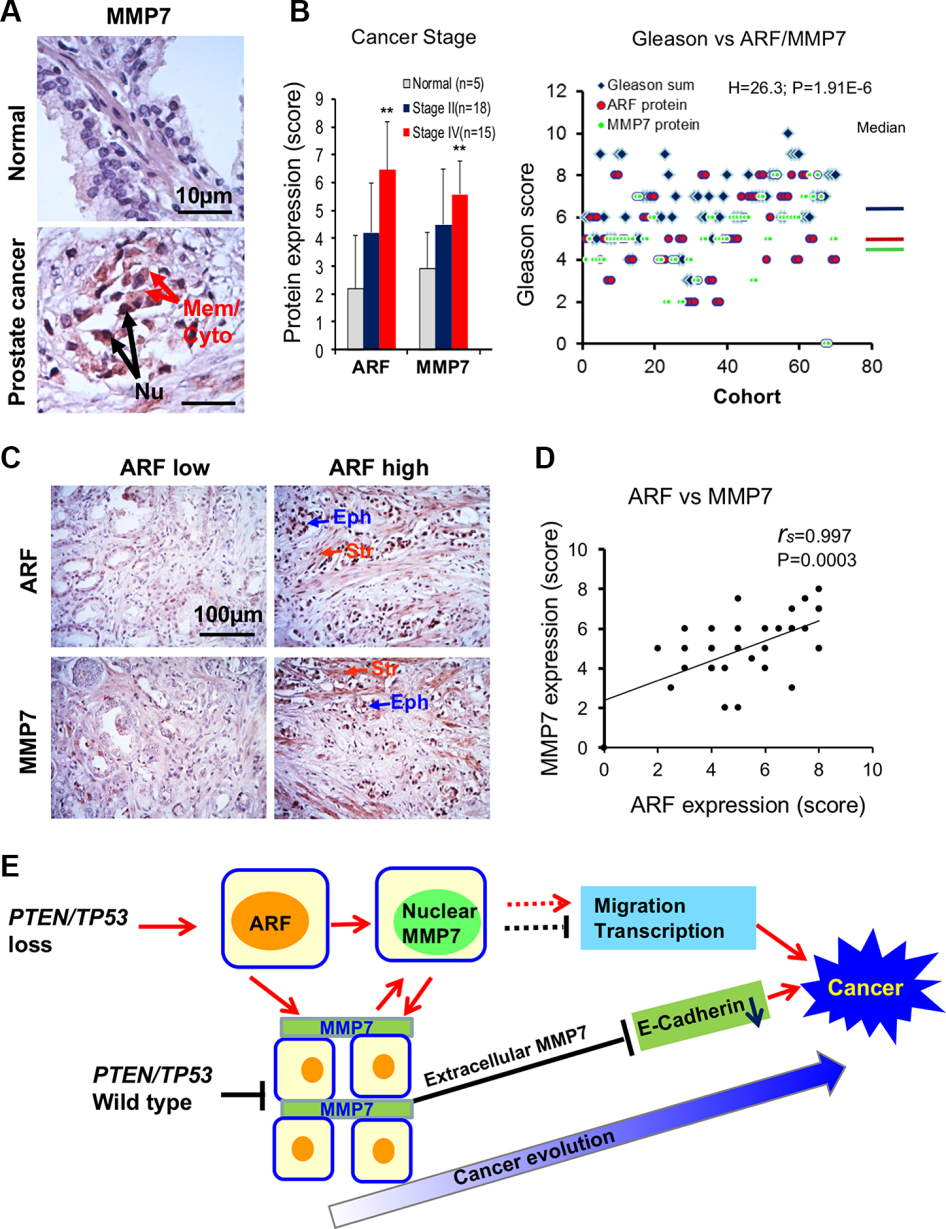
MMP7 correlates with ARF in human prostate cancer specimens (**A**) IHC staining reveals that MMP7 levels are increased in prostate cancer specimens as compared to normal tissues. Arrows indicate MMP7 expression on the membrane (Mem), the cytoplasm (Cyto) or the nucleus (Nu) of prostate cancer samples. (**B**) Levels of ARF and MMP7 expression among normal, stages II and IV of prostate cancer. Student's *t*-test was used to compare the expression levels at different stages. Kruskal-Wallis H test was used to determine the correlations between Gleason scores and expression levels of ARF/MMP7. (**C**) IHC staining of ARF and MMP7 in epithelial and stromal cells of human prostate cancer specimens. Eph: epithelial cells, Str: stromal cells. (**D**) A significant correlation between ARF and MMP7 expression in prostate cancer specimens. Spearman's coefficient method was applied to determine the correlation. (**E**) Signaling networks of PTEN/TP53, ARF, MMP7, and E-Cadherin for prostate cancer progression and evolution.

Given the decisive role of ARF and MMP7 interactions on tumor progression *in vitro* and *in vivo*, we examined the correlation between MMP7 and ARF in human PCa specimens. We performed IHC staining of MMP7 and ARF in PCa tissue microarrays (TMA) containing various stages and Gleason scores of primary cancer specimens. Notably, MMP7 was detected primarily in malignant cells of cancer lesions, with the intensive staining at the cytoplasm and plasma membrane (Figure 5A, arrows indicated). Most importantly, elevated MMP7 was accumulated in the nucleus of malignant cells (Figure 5A). MMP7 was detected barely in luminal epithelial cells of normal tissues, although the weak staining was observed at the cytoplasm and plasma membrane of basal cells. Similar to ARF, MMP7 levels were significantly elevated in advanced stages (IV) and high Gleason scores of PCa as compared to that in stages II PCa with low Gleason scores and normal tissues (Figure 5B). Similar to MMP7, ARF elevation was found in both epithelial and stroma cells of PCa specimens (Figure 5C). MMP7 levels were significantly correlated with ARF in PCa samples (Figure 5D, *r*_s_ = 0.997, *P* < 0.001). Thus, our data demonstrated that ARF may increase MMP7 levels to boost tumor microenvironments in human advanced PCa. In summary, our results indicate that ARF may promote cell motility and cancer progression through upregulation of MMP7 upon PTEN/TP53 inactivation.

## DISCUSSION

In this study, we elucidated *in vitro* and *in vivo* a novel mechanism in which ARF-MMP7 network contributes to PCa through enhancement of tumor microenvironments, at least in context of PTEN/TP53 loss. Moreover, the concomitant elevation of MMP7 and ARF in nucleus is associated with the malignancy of cancer cells. The nuclear localizations of MMPs have been reported in cancers, but the molecular mechanism underlying this phenomenon remains elusive [27, 28]. We demonstrated that ARF tumor suppressor stimulates the nuclear shuttling of MMP7 in PCa cells during cancer evolution. Our results provide some mechanistic explanations on the pro-proliferative and oncogenic-promoting roles of ARF in cancer cells [9, 15]. Collectively, the non-canonical ARF signaling may be more complicate than thought as it may be involved in ECM and EMT network in cancers.

PCa diseases result from the reprogramming of normal prostatic cells into invasive adenocarcinoma with metastasis potentials upon oncogenic insults and genetic alterations. MMP7 is associated with cell proliferation, mobility and cancer progression [19], yet the mechanism on MMP7 elevation in cancers is poorly understood. On the other hand, dysregulation of PTEN/PI3K pathways contributes to cancer progression through activation of downstream oncogenic pathways including MMP7, yet the mechanism on the aberrant elevation of MMP7 is puzzling [29]. Here we disclosure that MMP7 elevation induced by ARF potentiates EMT and tumor microenvironments for PCa progression using *Pten/Trp53* mouse models and human PCa cells. Since ARF elevation by PTEN loss activates EMT [16], our findings provide a logic link with more evidence on the oncogenic network of PTEN/PI3K/mTOR and ARF-MMP7-EMT pathways. This notion highlights a unique signaling pathway of PTEN, ARF and MMP7 and their oncogenic relevance in PCa (Figure 5E).

One intriguing phenomenon in this study is that ARF serves as a protein-binding docking of MMP7 in nucleus of PCa cells, and knockdown or inactivation of ARF abrogates nuclear MMP7 (Figures 2 and 4, and Supplementary Figure S2). Increasing evidence showed that, in addition to ECM, MMPs are also found in nucleus of cells, underscoring the pivotal roles of nuclear MMPs on cellular functions [18, 27, 30]. For example, nuclear MMP2 cleaves PARP in nucleus for the degradation to promote cell invasion [27, 31]. Nuclear MMP3 activates CCN2 gene transcription for IL-6-mediated cell migration [28, 32]. As MMP7 is associated with the malignant features of cancers [33, 34], our findings offer novel and valuable insights into understanding the functions of MMP7 in PCa as following: 1) MMP7 shuttles to nucleus without being cleaved, and it certainly deserves further investigation to elucidate the mechanisms on shuttling and the biological actions of MMP7 in nucleus of PCa cells; 2) ARF binds pro-MMP7 (~30 kDa) not active-MMP7 (~19 kDa) in nucleus, and it will be interesting to know these binding domains of MMP7 and ARF proteins; 3) Nuclear factors associated with ARF-MMP7 in nuclear compartments promote cell migration of PCa cells. ARF knockdown decreases MMP7 at both the transcription and the protein stability (Supplementary Figures S3 and S4), suggesting the ARF-MMP7 binding in nucleus may prevent pro-MMP7 from degradation. In addition to the NLS partially conserved in other MMPs [18] (data not shown), MMP7 protein contains a SUMOylation site at K64 as predicted by SUMOplot™ (www.abgent.com/sumoplot). As ARF determines SLUG stability in nucleus through SUMOylation [16], ARF may couple with several nuclear proteins to promote the SUMOylation-mediated stability of MMP7 in nucleus. Therefore, it is likely that MMP7 imports and accumulates in nucleus via a mechanism independent of NLS for stabilization after ARF-mediated SUMOylation.

In summary, our findings highlight a novel mechanism of ARF-MMP7 signaling in which ARF elevation promotes cell invasion through increasing oncogenic potentials of MMP7 for E-Cadherin downregulation in PCa progression and evolution (Figure 5E). Given that MMP7 is a potential “druggable” target on cancers in clinics, mechanisms on ARF-MMP7 inhibition would produce valuable information on the development of effective therapeutic treatments. A combined targeting of ARF and MMP7 would be of great significance in treatment of advanced PCa.

## MATERIALS AND METHODS

### Mutant mice and tumor analysis

Mice were bred and maintained in accordance with IACUC guidelines at Meharry Medical College. *Pten* and *Trp53* conditional knockout in prostates are controlled by Probasin-Cre recombination [35]. Generation, genotyping and prostate tumor analysis of wild type (*Wt*), *Pten*^pc−/−^*; Trp53*^pc−/−^ double-mutant (referred to as *Pten/Trp53*), and *Pten*^pc−/−^*; Trp53*^pc−/−^*; p19*^Arf+/−^ as well as *Pten*^pc−/−^*; Trp53*^pc−/−^*; p19*^Arf−/−^ triple-mutant mice (referred to as *Pten/Trp53/p19*^Arf^) were performed as described previously [9, 16].

### Transcriptome microarray analysis

Total RNA was subject to gene expression profiling using the Affymetrix Mouse Gene 1.1-ST v1 whole transcriptome array (Affymetrix, Santa Clara, CA). Data analysis was performed at Meharry Bioinformatics Core (Nashville, TN) using Partek Genomics Suite version 6.6 (Partek Inc., St. Louis, MO). Affymetrix CEL files were normalized using the Robust Multi-array Average (RMA) algorithm [36, 37]. Fold changes of transcriptome levels and the significance analyses were calculated using ANOVA. Significantly changed transcripts were defined as having a ≥ ± 2.0 fold expression from controls and an ANOVA *P*-value ≤ 0.05.

### Tet-inducible cell lines, IGF treatment and cell migration assay

Tet-inducible cell lines were established by inserting p14^ARF^-GFP-IRES-Luciferase cassettes into the Tet-on system (Clontech). First, the Tet receptor (Tet-R) expressing plasmids were introduced into LNCaP or C4-2B cell lines and selected by zeocin antibiotics. Secondly, stable cell lines of Tet-R-LNCaP or Tet-R-C4-2B were transfected with plasmids containing p14^ARF^-GFP-IRES-Luciferase and selected by Puromycin. For IGF stimulation, 22Rv1 cells were treated with IGF at a concentration of 100 μg/ml for 6 hrs. For migration assay, cells were grown until 70–80% confluence followed by serum starvation for 40 hrs. Cells were seeded at a density of 5 × 10^4^/well for PC3 cells or 7.5 × 10^4^/well for LNCaP cells in serum-free medium into the upper chamber with 8 μm polyethylene terephalate membrane filters (Falcon cell culture insert, Becton-Dickinson). Cells were allowed to migrate for 24 hrs for PC3 cells or 48 hrs for LNCaP cells in a humidified chamber at 37°C with 5% CO_2_. Non-migrant cells on the upper side of the filters were removed using cotton swab. Filters containing cells were fixed with 4% formaldehyde for 15 min, and cells in the lower filter were stained with 0.5% crystal violet and counted from three microscopy fields.

### Western blotting and fractionation analysis

For Western blotting, cell lysates were prepared in RIPA buffer [1× PBS, 1% Nonidet P-40, 0.5% sodium deoxycholate, 0.1% SDS, and protease inhibitor cocktail (Roche)], and then were subject to standard procedures of SDS-PAGE and antibody detection [26]. Antibodies used were: β-actin (AC-74, 1:5000, Sigma), MMP7 (monoclonal, JL07, sc80825, 1:500, Santa Cruz), p14^ARF^ (14P02, 1:750, NeoMarkers), E-Cadherin (sc33743, 1:500, Santa Cruz). Cell fractionation was performed using NE-PER nuclear and cytoplasmic protein extraction kit or subcellular protein fractionation kit (Thermo Scientific) followed by standard procedures of SDS-PAGE and antibody detection for IB using primary antibodies to: MMP7 (monoclonal, JL07, sc80825, 1:500, Santa Cruz or ab176325, 1:1000, Abcam), PARP (9542, 1:1000, Cell Signaling), HSP90 (H114, sc-7947, 1:1000, Santa Cruz).

### Co-immunoprecipitation (Co-IP), immunofluorescence (IF) and ARF knockdown by shRNA

For Co-IP experiment, HEK293T cells were transfected with GFP-tagged MMP7 and Myc-tagged p14^ARF^ plasmids. Cell lysates were collected 48 hrs post-transfection in RIPA buffer (1XPBS, pH 7.4, 2 mM EDTA, 1% Triton X-100, 0.5% sodium deoxycholate, and protease inhibitor), and used for immunoprecipitation by incubating with anti-Myc or anti-GFP antibodies for 1hr in cold room [26]. The A/G plus agarose beads were used for immunoprecipitation of antibody-antigen complex, and IgG was used as a control. The reactions were stopped by adding SDS loading buffer, and samples were subject to Western blotting. For IF experiment, cells were grown on coverslips for 2 days and fixed for 15 min in ice-cold methanol. Cells on slides were probed with following antibodies: p14^ARF^ (14P02, 1:200, NeoMarkers), MMP7 (monoclonal, JL07, sc80825, 1:50, Santa Cruz) or MMP7 (polyclonal, AF907, 1:200, R & D system) overnight at 4°C, followed by incubation with Alexa Fluor 568 or 488 dye conjugated to anti-mouse or rabbit IgG (Invitrogen) antibodies. Slides were washed and mounted with Vectashield medium (Vector Laboratories), and images were scanned with confocal microscopy [38]. p14^ARF^ knockdown in PC3 cells were generated using shRNA as described previously [16, 26].

### Statistical analysis

Data were evaluated using two-tail student's *t*-test, the values of *P* ≤ 0.05 were considered statistically significant. In particular, to determine the significance of differences in human prostate specimens, data were evaluated using Kruskal-Wallis H test or Spearman's test as indicated in figure legends.

### Immunohistochemistry (IHC) and prostate tissue microarrays (TMA)

For IHC analysis, after mouse tissues were fixed and processed, sections were probed using primary antibodies: E-Cadherin (24E10, 1:400, Cell Signaling), MMP7 antibody (D4H5-XP, 3801, 1:50, Cell Signaling), or Ki-67 antibody (NCL-Ki-67p, 1:200, Novocastra). Human prostate tissue microarrays (TMA) containing PCa (*n* = 35) and normal tissues (*n* = 5) in duplicates (US Biomax) were used for IHC staining with antibodies of p14^ARF^ (4C6/4, 1:50, 2407, Cell Signaling) and MMP7 (JL07, sc80825, 1:50, Santa Cruz). The staining intensities were scored as previously described [39], and analyzed for the correlation between ARF and MMP7.

## SUPPLEMENTARY MATERIALS AND METHODS





## Abbreviations

PCa: prostate cancer
ECM: extracellular matrix
EMT: epithelial-mesenchymal transition
MMP: matrix metalloproteinase
IHC: immunohistochemistry
