# Supplementary material for: MMP7 interacts with ARF in nucleus to potentiate tumor microenvironments for prostate cancer progression in vivo
Source: Oncotarget. 2016 Jun 23;7(30):47609–19. doi: 10.18632/oncotarget.10251 (PMC5216965; doi:10.18632/oncotarget.10251)
Supplement: Supplementary file 1 [file oncotarget-07-47609-s001.pdf]

MMP7 interacts with ARF in nucleus to potentiate tumor microenvironments for prostate cancer progression *in vivo*

Supplementary Materials

SUPPLEMENTARY INFORMATION ON MATERIALS AND METHODS

Additional antibodies

Additional antibodies for IHC are Vimentin (D21H3, XP® Rabbit mAb 5741, Cell Signaling), Snail (C15D3, Rabbit mAb 3879, Cell Signaling), Slug (C19G7, Rabbit mAb 9585, Cell Signaling). Additional antibodies for Western blot are MMP7 (EPR1251(2), ab176325, Abcam) and MMP7 (MM0022-4C21, sc-101566, Santa Cruz).

Knockdown of MMP7 and cell migration assay

PC3 cells were transfected wtih siRNA of MMP7 oligos or scrambled control oligos (Dharmacon), and cell lysates were collected 48 hrs later for Western blot. At 48 hrs post transfection followed with serum starvation for 16 hrs, the scratch was performed on confluent cells in 24 well plates in triplicates using a 200 ul pipette tip. The distances of closure were recorded by microscope photography 24 hrs post scratch, and were measured for statistical analysis.

Real time quantitative PCR (qRT-PCR)

Quantitative qRT-PCR analysis was performed as previously described [1]. Briefly, total RNAs were extracted from PC3-Scrambled and PC3-shARF cells using TRIZOL reagent (Invitrogen). The total cDNAs were synthesized by reverse transcription with SuperScript III first-strand synthesis kit (Invitrogen). Real-time quantitative PCR was performed with a Bio-Rad CFX96 real-time system in triplicates in a 20 µl reaction volume consisting of 10 µl iQ SYBR Green Supermix (Bio-Rad), 3 µl cDNAs, 0.5 µM of each primer for each sample, using the MMP7 primers: sense, 5′-tgagctacagtgggaacagg-3′; and antisense, 5′-tcatcgaagtgagcatctcc-3′ [2] . The level of target mRNA was determined by comparative CT method (ΔΔCT).

Cycloheximide (CHX) chase assay

To analyze the effect of ARF knockdown on the stability of MMP7 protein, CHX chase assay was conducted according to the method described previously [3]. Briefly, PC3-Scrambled and PC3-shARF cells were treated with CHX at 50 µg/ml for 0, 1.5, 3, 5, 7, 23 hrs, and cell lysates were collected for Western blot.

REFERENCES

1. Lu W, Xie Y, Ma Y, Matusik RJ, Chen Z. ARF represses androgen receptor transactivation in prostate cancer. Mol Endocrinol. 2013; 27:635–48.
2. Ito TK, Ishii G, Chiba H, Ochiai A. The VEGF angiogenic switch of fibroblasts is regulated by MMP-7 from cancer cells. Oncogene. 2007; 26:7194–203.
3. Xie Y, Liu S, Lu W, Yang Q, Williams KD, Binhazim AA, Carver BS, Matusik RJ, Chen Z. Slug regulates E-cadherin repression via p19Arf in prostate tumorigenesis. Mol Oncol. 2014; 8:1355–64.

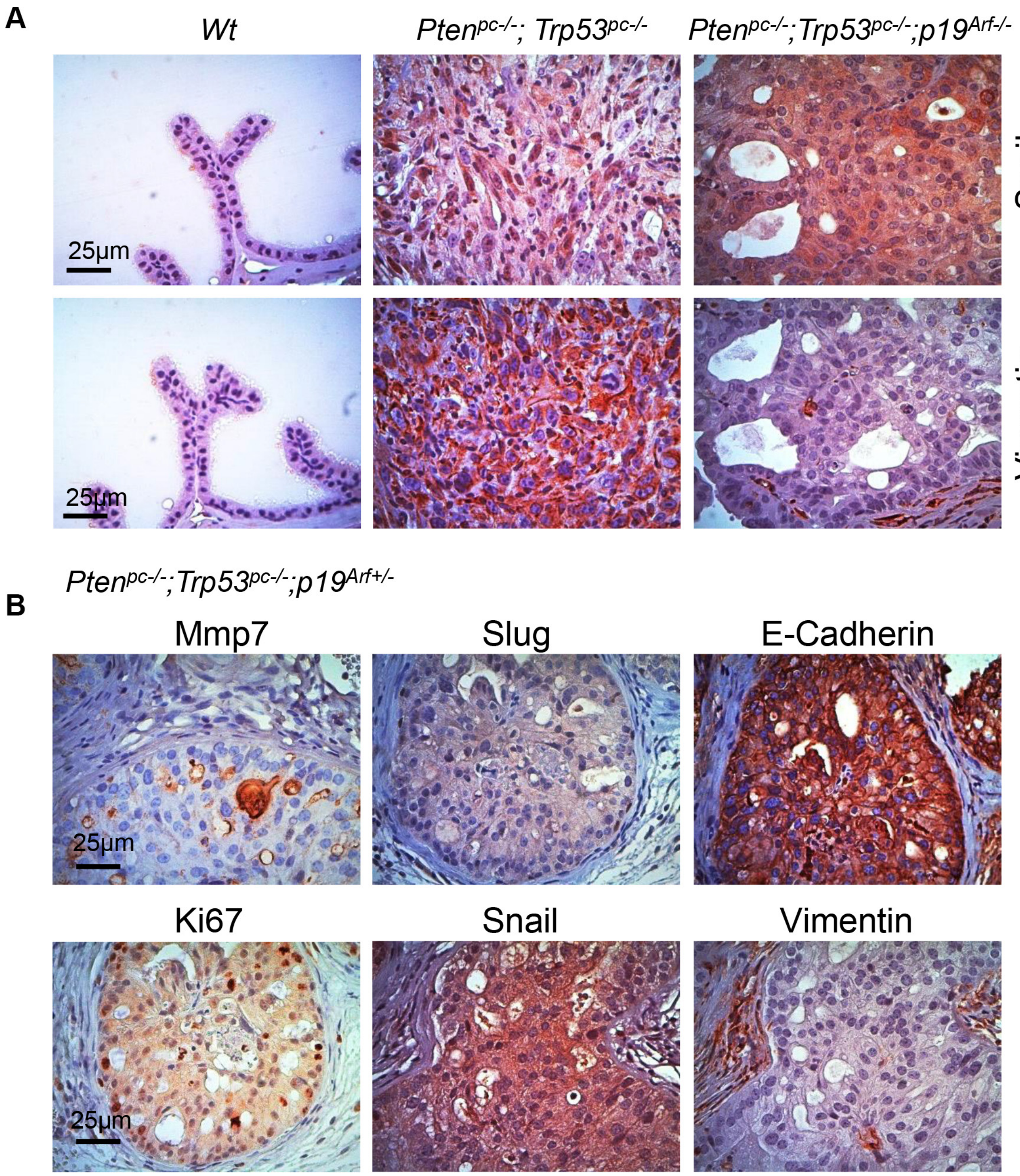

Supplementary Figure S1: p19Arf loss suppresses EMT in prostate tumors of *Pten/Trp53* mutant mice. (**A**) The expression levels of Snail and Vimentin proteins are decreased by $p19^{Arf}$ deficiency in mice. (**B**) Effects of loss of one allele of $p19^{Arf}$ on EMT markers in $Pten^{pc-/-}$; $Trp53^{pc-/-}$; $p19^{Arf+/-}$ mice. IHC staining of indicated proteins were performed using standard protocols as described in Materials and Methods.

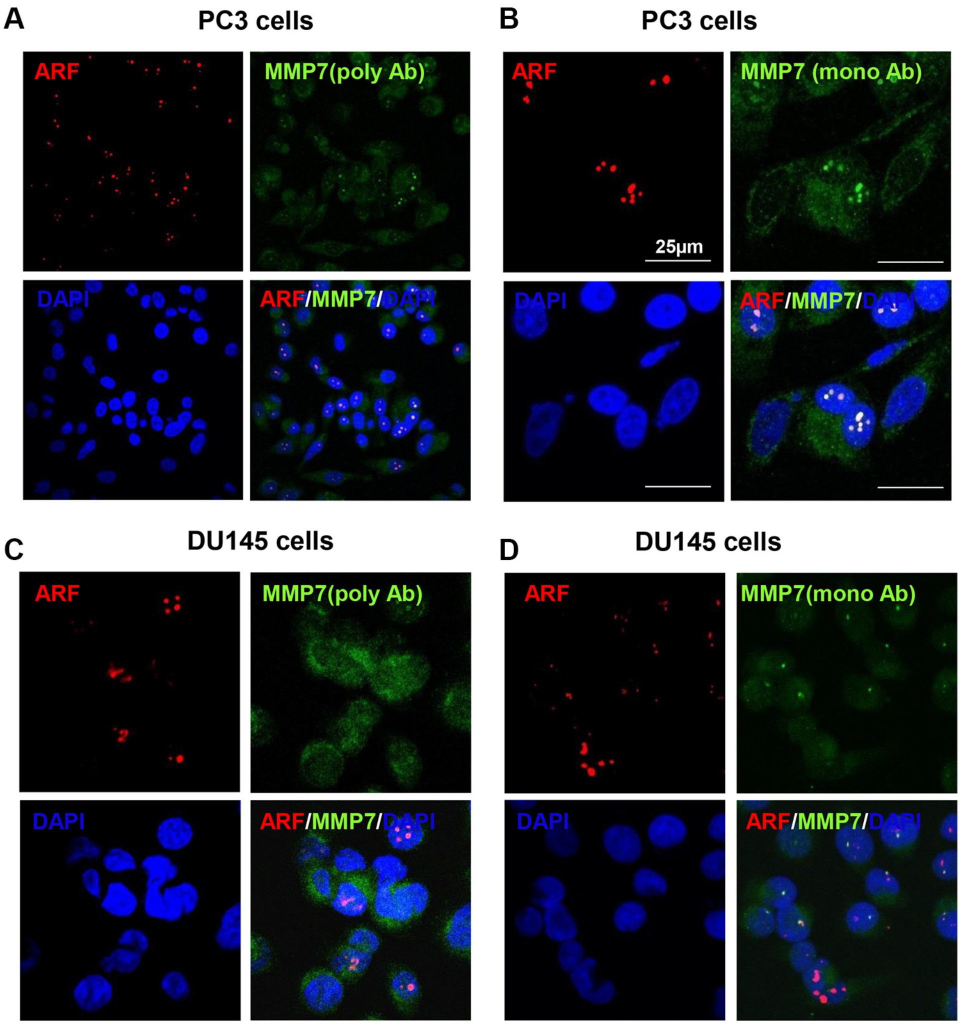

Supplementary Figure S2: The co-localization of ARF and MMP7 in PC3 and DU145 cells. IF experiments were performed using different MMP7 antibodies.

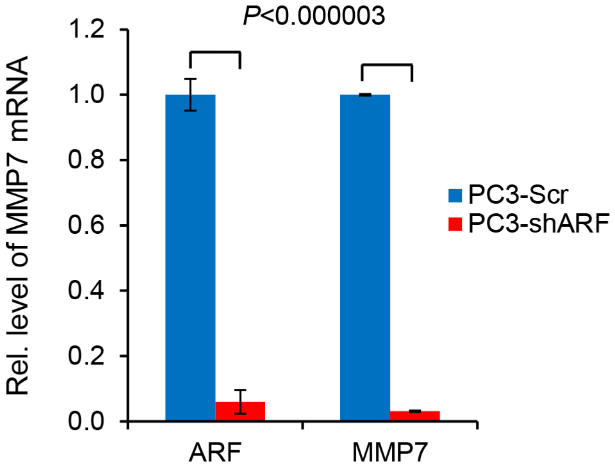

Supplementary Figure S3: ARF knockdown decreases the mRNA level of MMP7 in PC3 cells. Quantitative qRT-PCR analysis was performed as previously described (Lu et al. Mol. Endo 2013). The level of target mRNA was determined by comparative CT method (ΔΔCT). Error bars represent means ± SD of triplicates.

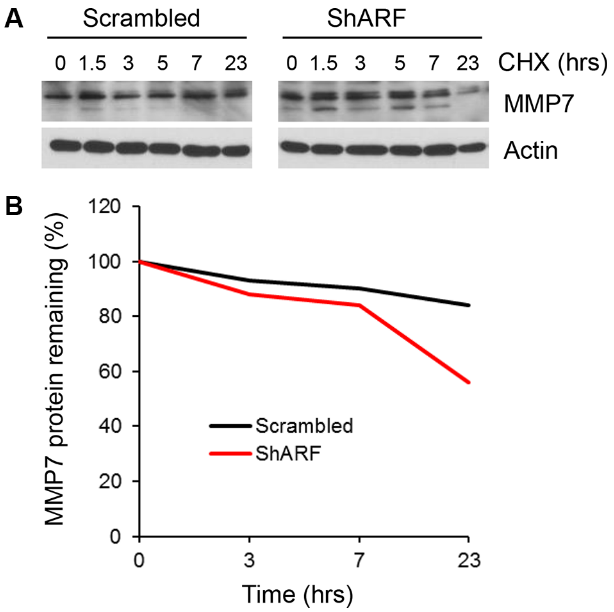

Supplementary Figure S4: ARF knockdown shortens the half-life of MMP7 protein. (**A**) Western blot analysis to show MMP levels in PC3 cells after CHX treatment. CHX chase experiments were performed with CHX treatment (50 µg/ml) at indicated times. (**B**) Quantification of protein bands in (A) using densitometry.

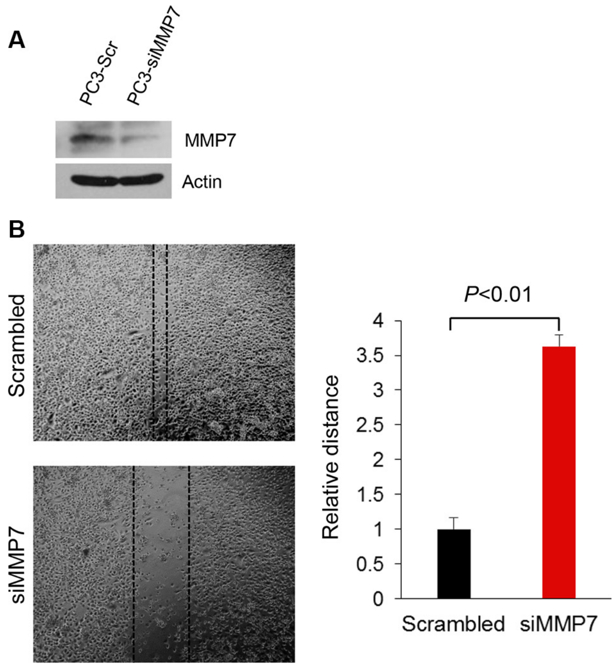

Supplementary Figure S5: MMP7 knockdown decreases cell migration in PC3 cells. (**A**) MMP7 knockdown was confirmed by Western blot analysis. MMP7 siRNA oligos or scrambled control oligos were transfected to PC3 cells, and cell lysates were collected for Western blot after 48 hrs. (**B**) The effects of MMP7 knockdown on cell migration. The distances of closure were recorded by microscope photography 24 hrs post scratch, and were measured for statistical analysis..

Supplementary Table S1: p19Arf regulated genes in *Pten/Trp53* and *Pten/Trp53/p19^{Arf}* mouse tumors using microarray analysis of differentially expressed transcriptome

Upregulated genes	Fold changes	Gene functions
Mmp7	12.5	proteolysis, metabolic process, collagen catabolic process, cell proliferation
Dmbt1	6.0	inner cell mass, cell proliferation, transport , multicellular organismal development, protein transport, cell differentiation, positive regulation of epithelial cell differentiation
Muc20	4.3	activation of MAPK activity, c-Met signaling pathway
Mmp15	4.1	proteolysis , metabolic process
**Downregulated genes**		
H2-M9	–4.4	biological process
Vipr2	–2.3	signal transduction, cell surface receptor linked signal transduction, G-protein coupled receptor, protein signaling pathway
Ogg1	–1.5	DNA repair, base-excision repair, nucleotide-excision repair, response to DNA damage stimulus, metabolic process

Supplementary Table S2: A complete list of genes with altered expression in mouse prostate tumors upon p19^{Arf} loss. See Supplementary_Table_S2
